# Interaction Between Sex and Cardiac Interoceptive Accuracy in Measures of Induced Pain

**DOI:** 10.3389/fpsyg.2020.577961

**Published:** 2021-02-09

**Authors:** Eszter Ferentzi, Mattis Geiger, Sandra A. Mai-Lippold, Ferenc Köteles, Christian Montag, Olga Pollatos

**Affiliations:** ^1^Institute of Health Promotion and Sport Sciences, ELTE Eötvös Loránd University, Budapest, Hungary; ^2^Department of Individual Differences and Psychological Assessment, Institute of Psychology and Education, Ulm University, Ulm, Germany; ^3^Department of Clinical and Health Psychology, Institute of Psychology and Education, Ulm University, Ulm, Germany; ^4^Department of Molecular Psychology, Institute of Psychology and Education, Ulm University, Ulm, Germany

**Keywords:** interoception, cardiac interoceptive accuracy, pain perception, affect, sex differences

## Abstract

Pain perception is influenced by several factors, and among them, affect, sex, and perception of bodily signals are assumed to play a prominent role. The aim of the present study is to explore how sex, cardiac interoceptive accuracy, and the interaction of the latter two influence the perception of experimentally induced pain. We investigated a large sample of young adults (*n* = 159, 50.9% female, age: 23.45, SD = 3.767), assessing current positive and negative affective state with the Positive and Negative Affect Schedule (both involved as control variables), cardiac interoceptive accuracy with the mental heartbeat tracking task, and pain sensitivity with electrical stimulation on the back of the dominant hand, applying a repeated-measures staircase protocol. Males showed a significantly higher pain threshold and tolerance level than females, whereas cardiac interoceptive accuracy was not associated with pain sensitivity. The impact of sex × cardiac interoceptive accuracy interaction was significant for pain threshold only, while pain tolerance was predicted only by sex. According to these findings, the associations between pain sensitivity, cardiac IAc, and sex might be more complicated than it was supposed in previous studies. Interactions between factors impacting pain perception appear worthy of further investigation.

## Introduction

Pain is defined as an unpleasant experience with sensory and emotional components, associated with potential tissue damage (Merskey and Bogduk, 1994), and strongly influenced by somatic, psychological, and social factors (Moseley, 2007). Neuroanatomical evidence demonstrates that pain processing shows a considerable overlap, with processing of visceral signals representing the actual homeostatic condition of the body; pain was also called the homeostatic emotion (Craig, 2003). Based on these findings, a broadperspective on interoception was proposed, which includes pain, itch, sensual touch, the sense of the metabolic state of the muscles, and other modalities beyond the classic visceroceptive channels (Craig, 2010). Concerning the conscious aspects of interoception, Garfinkel and colleagues (Garfinkel et al., 2015) proposed a multifaceted model, which differentiates objective (called interoceptive accuracy, IAc), subjective (interoceptive sensibility), and metacognitive (interoceptive awareness) dimensions of interoception. Although cardiac IAc cannot be generalized to other interoceptive modalities (Ferentzi et al., 2018), it is often used as an indicator of the acuity of perception of interoceptive signals. As the experience of pain is accompanied by a marked cardiovascular response (Gracely, 1999), the use of cardiac IAc is well justified in this area of research.

Both interoception and pain are interpreted as sensitivity towards bodily or body-related signals, associated with cognitive and emotional regulatory processes (Craig, 2003, 2015); thus, a connection between the two constructs seems plausible. However, the relationship of cardiac IAc and pain is not yet well understood, as empirical evidence provided so far is contradictory.

A systematic review drew the conclusion that patients with chronic pain syndromes are characterized by a less accurate perception of heartbeats than healthy control subjects (Di Lernia et al., 2016). Chronicity of the condition, however, might play an important role in theseresults. Studies that investigated non-clinical samples either found that more accurate heartbeat perception (as assessed by the mental heartbeat tracking task; Schandry, 1981) was associated with decreased pain threshold and tolerance (Pollatos et al., 2012; Weiss et al., 2014) or revealed no association (Werner et al., 2009; Ferentzi et al., 2018). It is an open question what might explain these contradictory findings. Pollatos et al. (2012) investigated 60 participants and found a correlation of medium effect size (cardiac IAc and pain threshold: *r* = −0.42; *p* < 0.01; cardiac IAc and pain tolerance: *r* = −0.33, *p* < 0.05) similarly to Weiss et al. (2014; *n* = 30, cardiac IAc and pain threshold: *r* = −0.53; *p* < 0.01; cardiac IAc and pain tolerance: *r* = −0.44, *p* < 0.05); both studies used a pressure algometry to induce pain. Both the studies of Weiss et al. (2014) and Werner et al. (2009) used preselected samples. The former investigated a clinical sample as well, while the latter matched samples of people with high and low heartbeat perception (*n* = 62). The two studies that found no association applied heat (Werner et al., 2009) or induced ischemic pain (Ferentzi et al., 2018). The diversity of the applied methodologies could partly influence the incoherence of the findings.

A candidate to help understanding the pain and cardiac IAc relationship is sex because it seems to relate to both pain perception and cardiac IAc. Males usually show higher pain threshold and tolerance level (Rhudy and Williams, 2005; Fillingim et al., 2009) as well as higher cardiac IAc (Whitehead et al., 1977; Jones and Hollandsworth, 1981; Katkin et al., 1981; Katkin, 1985; Harver et al., 1993; Montoya et al., 1993; Grabauskaitė et al., 2017). Interestingly, there is no agreement concerning the explanation of the latter finding. Body composition might be an influencing factor (Montgomery et al., 1984; Jones et al., 1987) and could be in the background of sex differences in IAc (Rouse et al., 1988). There is a study investigating children, but it still found differences between males and females even after controlling for body mass index (Koch and Pollatos, 2014). Cardiodynamic characteristics (e.g., stroke volume and the momentum of ejected blood mass; Schandry et al., 1993; Khalsa et al., 2009) may also lie behind sex differences in heartbeat perception. Moreover, the existence of sex difference in cardiac IAc is not uniformly supported by empirical findings; there are studies that reported no divergence between sexes (e.g., Mussgay et al., 1999; Pollatos and Schandry, 2004).

Unlike cardiac IAc, the difference between males and females in pain sensitivity is widely supported by empirical studies (Fillingim et al., 2009). According to recent findings, females show higher pain sensitivity than males independently from the applied experimental pain evoking modality (Ostrom et al., 2017). Sex differences in pain perception have been explained by various modulating psychological factors (Ostrom et al., 2017); however, differences in cardiac IAc or other interoceptive modalities have not been mentioned among these. In summary, males are characterized by higher cardioceptive IAc and lower pain sensitivity than females. This might indicate that sex plays a moderating role on the association between interoception and pain sensitivity.

Furthermore, according to theoretical accounts and empirical investigations, both pain perception and cardiac IAc are tightly linked to affect. In the case of pain, it is not clear which aspect of affect is relevant. On the one hand, most studies investigating the effect of experimentally induced emotions found that pleasant affective states are associated with higher pain threshold and tolerance, and negative states with increased sensitivity toward pain (Meagher et al., 2001; Carter et al., 2002; Villemure et al., 2003; Rainville et al., 2005). According to other findings, on the other hand, the emotion stimulation itself (along with further modifying factors, such as attention) is associated with reduced pain sensitivity, while the emotional valence of the stimulation is less relevant (Arntz et al., 1991; Arntz and de Jong, 1993; Villemure, 2002; de Tommaso et al., 2009). Interoception, in general, is believed to be tightly linked to emotional experience (James, 1884; Cannon, 1927; Damasio, 2010). Recent empirical studies applying emotional stimuli seem to confirm this view (e.g., Herbert et al., 2007, 2010; Pollatos et al., 2007a). There is evidence that the spontaneous affective state is also related to interoception. For example, state anxiety is associated with higher cardiac IAc as assessed by mental heartbeat tracking task (Schandry, 1981; Ludwick-Rosenthal and Neufeld, 1985; Pollatos et al., 2007b). Thus, controlling for the affective state might shed more light on the relationships between sensitivity to pain, cardiac IAc, and sex.

The aim of the present study was to better understand how current affective state, heartbeat perception ability, and sex influence pain perception in healthy individuals. Our hypotheses are that higher cardiac IAc is associated with lower (1) pain threshold and (2) pain tolerance. Furthermore, sex differences with respect to pain sensitivity were also assumed; we expected (3) higher pain threshold and (4) tolerance for males than females and (5) an interaction between sex and cardiac IAc on measures of pain.

## Materials and Methods

### Participants

A total of 159 university students (50.9% female, mean age: 23.45, SD = 3.767) participated in the study. They were compensated with 20€. The exclusion criteria included psychiatric or neurological illnesses with current, not treated symptoms. The participants were fluent in German and were recruited from a local database called Ulm Gene Brain Behavior Project. Participation was voluntary and anonymous. All participants signed a written informed consent before the start of the measurements. Ethical approval for the study was obtained from the ethics committee of Ulm University, Ulm, Germany.

As data collection was part of a larger study (see the details below), *a priori* sample size calculation was not possible. However, the minimum required sample size for linear multiple regression with five tested predictors and medium effect size (*f*^2^ = 0.15) is *n* = 138 (α = 0.05, power = 0.95; calculated with G^∗^Power v3.1.9.4. software, Faul et al., 2007). As the sample size of the present study exceeds this threshold, it can be considered appropriate for testing the hypotheses introduced above. We assumed medium effect size based on the study of Pollatos et al. (2012); this study investigated a similar sample and reported a correlation of *r* = −0.42 (*p* < 0.01) between IAc and pain threshold and *r* = −0.33 (*p* < 0.05) between cardiac IAc and pain tolerance.

### Study Protocol

The data collection started with the Positive and Negative Affect Schedule (PANAS) questionnaire (Watson et al., 1988). After that, all the electrodes were placed (measuring electrocardiogram, electromyogram, and skin conductance—the data of the latter two are not used here) using a portable BIOPAC system, Biopac MP150 system with the modules UIM100C, STP100C, ECG100C, GSR100C, and two EMG100C modules. Data were acquired with AcqKnowledge 4.1.1 (BUILD: 04.23.2010; 07.14.2010c; DISK46). This was followed by the heartbeat tracking task and the pain perception task. The data collection occurred as part of a larger study. The results of the measurements that followed are not reported here (i.e., pain regulation task, cognitive tasks, and emotion expression task).

### Measures

#### Positive and Negative Affect

The PANAS aims to assess positive (PA) and negative (NA) emotional states (Watson et al., 1988). We used the 20-item-long version of the PANAS that measures the current affective state of the participants, with 10 items per each affect (Watson et al., 1988). High scores refer to high level of affect. The internal consistencies assessed by Cronbach’s alpha were α = 0.84 (PA) and 0.83 (NA).

#### Cardiac Interoceptive Accuracy

Cardiac IAc was measured with the mental tracking method (Schandry, 1981) over four different periods of time with various length (25, 35, 45, and 60 s) presented in random order. The participants were seated comfortably, with palms facing up on a table in front of them. They were asked to count their heartbeats silently while not using any extra tricks to feel their heartbeats better. The participants were encouraged to report any number of heartbeats they could feel by emphasizing that there are no right and wrong answers and by adding that there are not many people who can feel their heartbeats correctly. If they were uncertain or could not feel their heartbeats, they were encouraged to still try to count or estimate the number of heartbeats. The accuracy score was calculated for each trial using the following formula: 1 – | (recorded heartbeats–counted heartbeats)/recorded heartbeats|; the measure of cardiac accuracy score was then calculated as an aggregate mean score over all items, ranging between 0 and 1. Thus, higher values indicate better IAc. Cronbach’s α for the four trials is 0.95 in the current study.

#### Pain Perception

Pain-related perception was measured with a psychophysical upward staircase threshold estimation task characterized by two thresholds: the pain threshold (when the slightest pain, i.e., more than anunpleasant sensation was felt for the first time) and the pain tolerance (when pain was not tolerable anymore). Pain stimuli were applied *via* electrical stimulation on the back of the dominant hand; the cathode was applied to the middle phalanx of the index and the anode to the proximal phalanx of the second finger (400 V; with a starting point of 0.1 mA, increasing by 0.1 mA with each trial to set the sensation threshold first and by 0.5-mA steps to measure pain threshold and tolerance). The length of each trial was 5 s of stimulation, with a 6–10-s-long break in between.

The participants were instructed that the aim of the study is to assess their sensitivity to electrical impulses. They were also informed that they will feel pain at a certain point of the assessment. In the case of pain threshold, they were asked to report when they feel the smallest signs or sensations of pain; in case of pain tolerance, they were asked to report when they cannot bear the pain anymore and want to interrupt the stimulation.

The participants verbally indicated when a stimulus crossed the currently assessed threshold. The stimulus intensity (in mA) rated as crossing the threshold (first painful or maximally tolerable stimulus) was taken as a threshold value. Stimulation was immediately interrupted if the participants reported that it crossed the tolerance threshold.

Pain threshold was assessed three times (average correlation between the three values is rho = 0.955, *p* < 0.001). The assessment re-started at the lowest threshold, minus three steps (0.3 or 1.5 mA). Pain tolerance was assessed two times (rho = 0.975, *p* < 0.001). The final score for each threshold was the average of the respective trials.

### Data Analyses

Statistical analysis was performed with JASP software 0.9.1 (JASP Team, 2019). According to the Shapiro–Wilk test of normality, only PA was normally distributed (*p* = 0.354; for all the other variables, *p* < 0.001). Therefore, Spearman correlations were computed to assess the associations between the variables. As sex has a strong influence on subjective pain ratings, sex differences were investigated separately with Mann–Whitney *U*-test. Effect sizes (ES) were estimated as a rank–biserial correlation. As the frequentist approach to statistical hypothesis testing has recently received criticism (Dienes, 2011; Jarosz and Wiley, 2014), hypotheses were tested using both frequentist and Bayesian methods.

Based on visual inspection, five outlier values were identified for both pain perception values at the trial level (from two participants); these values were winsorized, i.e., the original values were replaced by the nearest value from the same variable set.

To analyze the effect of positive and negative affective state, heartbeat perception score, and sex on pain perception, both frequentist and Bayesian regression analyses were conducted. In the frequentist approach, two different linear regression analyses were conducted for pain threshold and pain tolerance. The variables were entered using the ENTER method in four steps: (1) positive and negative affect, (2) sex (males = 1, females = 2), (3) heartbeat perception score, and (4) sex × heartbeat perception score interaction term. The interaction term was calculated as the product of the centered values of sex and heartbeat perception scores.

In the Bayesian approach, the null model always included positive and negative affect (see the details below). The results are presented as Bayes factors (BF_10_), showing the likelihood of the alternative hypothesis as compared to the null hypothesis. BF_10_ between 1 and 3 represent anecdotal, between 3 and 10 represent substantial, between 10 and 30 represent strong, between 30 and 100 represent very strong, and >100 represent decisive evidence in favor of the alternative hypothesis (Jeffreys, 1998; Jarosz and Wiley, 2014).

## Results

### Descriptive Statistics and Correlation Analyses

Table 1 presents the descriptive statistics and the correlations of the measured variables, while Table 2 presents the findings of the Bayesian correlation analysis. In summary, the results of both frequentist and Bayesian analyses indicate a weak positive correlation between positive affect and pain tolerance. The frequentist approach also shows a weak association between positive affect and pain threshold, whereas associations between pain sensitivity and negative affect were not supported. In fact, BF_10_ values indicated the superiority of the null hypothesis (i.e., the lack of association with positive and negative affect) for both tolerance and threshold. There is also a weak positive correlation between pain tolerance and cardiac IAc according to the frequentist analysis.

**TABLE 1:** Descriptive statistics (mean, standard deviation and minimum-maximum values in the diagonal list) of the assessed variables and Spearman correlation coefficients (with rho and *p* values); the last two columns present the data divided by sex.

*N* = 159	PA	NA	Cardiac IAc	pain th.	pain tol.	Male, *N* = 78	Female, *N* = 81
PA	31.51 ± 5.808 (17–47)	0.010 (0.903)	0.112 (0.158)	0.162 (0.042)	0.244 (0.002)	32.94 ± 5.957 (17–47)	30.14 ± 5.344 (19–46)
NA		13.21 ± 3.845 (10–35)	0.002 (0.979)	−0.064 (0.421)	−0.053 (0.511)	12.91 ± 3.358 (10–26)	13.50 ± 4.262 (10–35)
Cardiac IAc			0.70 ± 0.182 (0.23–0.98)	0.116 (0.147)	0.194 (0.014)	0.73 ± 0.182 (0.23–0.98)	0.67 ± 0.178 (0.29–0.96)
pain th.				2.31 ± 1.455 (0.50–6.50)	0.641 (<0.001)	2.82 ± 1.626 (0.50–6.50)	1.82 ± 1.070 (0.50–5.33)
pain tol.					6.03 ± 3.174 (1.00–17.25)	7.86 ± 3.343 (1.00–17.25)	4.27 ± 1.651 (1.50–8.50)

*Two-tailed in all cases. PA, positive affect; NA, negative affect; IAc, interoceptive accuracy; pain th., pain threshold (in mA); pain tol., pain tolerance (in mA).*

**TABLE 2:** Results of the Bayesian correlation analysis (Kendall’s tau-b coefficients).

*N* = 159	Negative affect	Heartbeat perception score	Pain threshold (mA)	Pain tolerance (mA)
Positive affect	0.007	0.083	0.117	0.171*
Negative affect		–0.003	–0.043	–0.036
Heartbeat perception score			0.072	0.132
Pain threshold (mA)				0.485***

*BF_10_ > 10; ***BF_10_ > 100.

### Sex Differences in Pain Measures

Significant sex differences with respect to both assessedpain-related variables were found. The males showed significantly higher pain threshold (*W* = 4,342, *p* < 0.001, d = 0.374) and pain tolerance level (*W* = 5,401, *p* < 0.001, d = 0.710) than the females. The Bayesian analysis indicated decisive evidence both for pain threshold and tolerance (BF_10_ = 123 and BF_10_ = 69511, respectively) in favor of the alternative hypotheses.

### Interaction Between Sex and Cardiac IAc on Pain Threshold and Pain Tolerance

**FIGURE 1:**
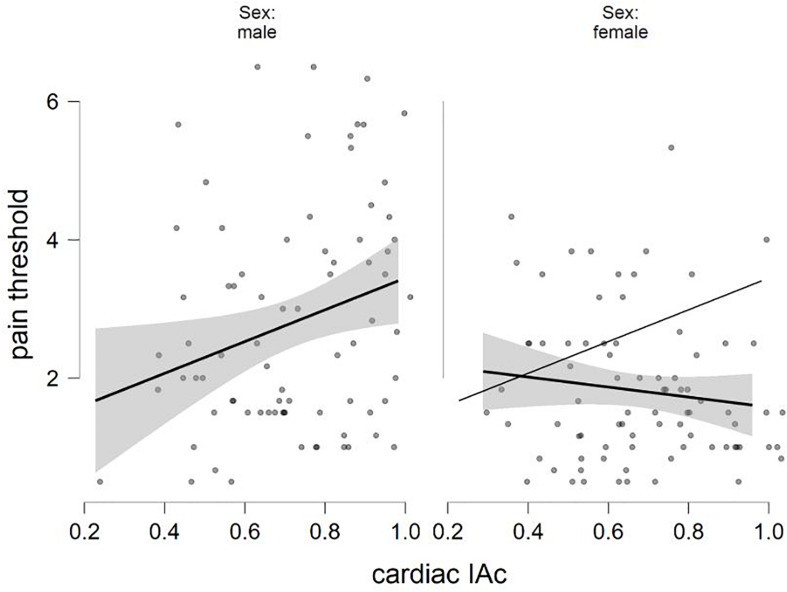
Negative interaction between sex and cardiac IAc in the prediction of pain threshold (fitted regression lines with confidence intervals).

The final equation of the linear regression analysis with pain threshold as criterion variable was significant [*F*(5,153) = 6.214, *p* < 0.001, *R*^2^ = 0.169], with sex (β = −0.324, *p* < 0.001; males showing higher values) and sex × cardiac IAc interaction (β = −0.192, *p* = 0.014) as significant predictors (see details in Table 3). Concerning the interaction, higher levels of heartbeat tracking ability were associated with disproportionately lower levels of pain threshold in females compared to males. Figure 1 shows how cardiac IAc relates to pain threshold by gender. The ghost line on the right shows the fitted regression line made for males. Note that the fitted lines of the figure do not consider the impact of positive and negative affect.

**TABLE 3:** Results of multiple linear regression analysis with pain threshold as criterion variable.

*N* = 159		Positive affect	Negative affect	Sex	Heartbeat tracking score	Sex × heartbeat tracking score
Step 1 *p* = 0.114 *R*^2^ = 0.027	B ± SE	0.033 ± 0.020	−0.036 ± 0.030			
	95.0% CIs for B	−0.006. 0.072	−0.095; 0.023			
	Standardized β	0.133	−0.095			
	*P*	0.095	0.232			
Step 2 *p* < 0.001 *R*^2^ = 0.126	B ± SE	0.014 ± 0.019	−0.027 ± 0.029	−0.943 ± 0.225		
	95.0% CIs for B	−0.025;0.052	−0.084; 0.029	−1.387; −0.498		
	Standardized β	0.055	−0.072	−0.325		
	*P*	0.480	0.338	< 0.001		
Step 3 *p* < 0.001 *R*^2^ = 0.135	B ± SE	0.012 ± 0.019	−0.027 ± 0.028	−0.905 ± 0.227	0.763 ± 0.609	
	95.0% CIs for B	−0.026;0.050	−0.084; 0.029	−1.352; −0.457	−0.440; 1.965	
	Standardized β	0.048	−0.072	−0.312	0.095	
	*P*	0.538	0.337	<0.001	0.212	
Step 4 *p* < 0.001 *R*^2^ = 0.169	B ± SE	−0.003 ± 0.020	−0.030 ± 0.028	−0.940 ± 0.223	0.762 ± 0.599	−3.111 ± 1.249
	95.0% CIs for B	−0.042; 0.037	−0.085; 0.025	−1.382; −0.499	−0.421; 1.945	−5.579; −0.643
	Standardized β	−0.010	−0.080	−0.324	0.095	−0.192
	P	0.899	0.283	<0.001	0.205	0.014

*B, unstandardized beta; SE = standard error for the unstandardized beta.*

**FIGURE 2:**
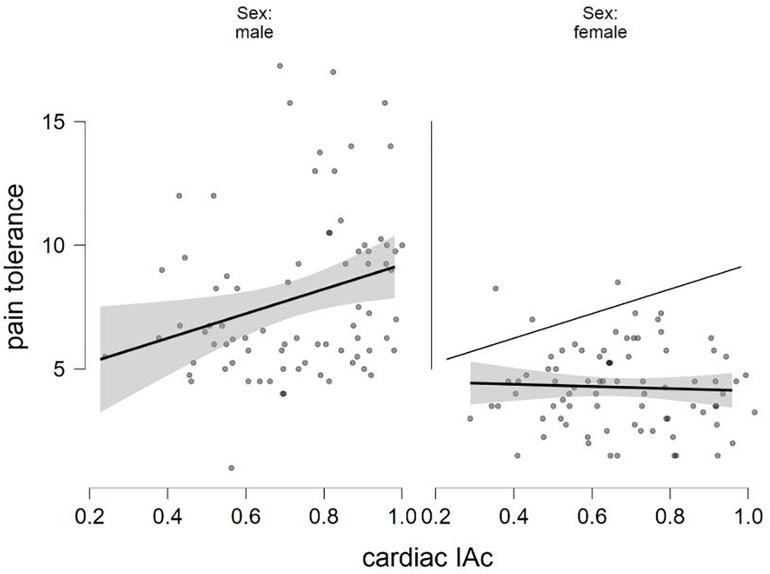
The relation of sex and cardiac IAc in the prediction of pain tolerance (fitted regression lines with confidence intervals).

The final equation predicting pain tolerance was also significant[*F*(5,153) = 18.354, *p* < 0.001, *R*^2^ = 0.375]. The males were again characterized by higher values (β = −0.519, *p* < 0.001). The interaction term was just above the accepted level of statistical significance (β = −0.126, *p* = 0.061), just like the cardiac IAc scores (β = 0.120, *p* = 0.067; Table 4). Figure 2 shows how cardiac IAc relates to pain threshold by gender. (Similar to Figure 1, the ghost line on the right shows the fitted regression line made for males. Note also that the fitted lines of the figure do not consider the impact of positive and negative affect. The cardiac IAc and indicators of the current affective state were not associated with indicators of pain sensitivity.

**TABLE 4:** Results of multiple linear regression analysis with pain tolerance as criterion variable.

*N* = 159		Positive affect	Negative affect	Sex	Heartbeat tracking score	Sex × heartbeat tracking score
Step 1 *p* < 0.001 *R*^2^ = 0.086	B ± SE	0.150 ± 0.042	−0.076 ± 0.063			
	95.0% CIs for B	0.067; 0.233	−0.201; 0.049			
	Standardized β	0.274	−0.092			
	*P*	<0.001	0.230			
Step 2 *p* < 0.001 *R*^2^ = 0.346	B ± SE	0.081 ± 0.037	−0.046 ± 0.054	−3.340 ± 0.425		
	95.0% CIs for B	0.009; 0.153	−0.152; 0.060	−4.178; −2.501		
	Standardized β	0.148	−0.056	−0.528		
	*P*	0.029	0.391	<0.001		
Step 3 *p* < 0.001 *R*^2^ = 0.360	B ± SE	0.076 ± 0.036	−0.046 ± 0.053	−3.235 ± 0.425	2.093 ± 1.142	
	95.0% CIs for B	0.004; 0.148	−0.152; 0.059	−4.075; −2.395	−0.162; 4.349	
	Standardized β	0.139	−0.056	−0.511	0.120	
	*p*	0.038	0.387	<0.001	0.069	
Step 4 *p* < 0.001 *R*^2^ = 0.375	B ± SE	0.055 ± 0.038	−0.050 ± 0.053	−3.286 ± 0.423	2.092 ± 1.132	−4.459 ± 2.363
	95.0% CIs for B	−0.019; 0.130	−0.155; 0.054	−4.121; −2.451	−0.145; 4.329	−9.124; 0.212
	Standardized β	0.101	−0.061	−0.519	0.120	−0.126
	*p*	0.145	0.345	<0.001	0.067	0.061

*B, unstandardized beta; SE, standard error for the unstandardized beta.*

In the Bayesian approach, when the null model included positive and negative affect and the alternative model included sex, the Bayes factor was BF_10_ = 539.991 for pain threshold and BF_10_ 1.075e + 10 for pain tolerance (which means that there is decisive evidence in favor of the alternative model). When sex was also added to the null model and the alternative model contained cardiac IAc, BF_10_ = 0.567 for pain threshold and BF_10_ = 0.912 for pain tolerance. In other words, the analyses were inconclusive with respect to the superiority of the null and the alternative model. The regression model predicting pain threshold that included sex × heartbeat perception score interaction term was almost five times more likely (BF_10_ = 4.695, indicating substantial evidence in favor of the alternative model) as the null model (including positive and negative affect, sex, and heartbeat perception score). In the case of pain tolerance, however, the alternative model was not more likely to occur (BF_10_ = 1.092). Thus, the Bayesian regression analyses supported the results of the frequentist analyses regarding the interaction term.

## Discussion

This study investigated the associations between sex and cardiac interoceptive accuracy with measures related to pain perception, i.e., pain threshold and pain tolerance, in 159 young individuals. After controlling for negative and positive affective state, the interaction between sex and cardiac IAc was a significant predictor of pain threshold, but not for pain tolerance.

The current affective state shows no consistent associations with pain sensitivity, indicating that the natural levels of positive and negative affect do not influence the sensitivity to experimentally induced pain in young individuals. The correlation between positive affect and pain tolerance, however, partly supports the view mentioned in the introduction, namely, that pleasant affective states are associated with lower pain sensitivity (Meagher et al., 2001; Carter et al., 2002; Villemure et al., 2003; Rainville et al., 2005). On the other hand, negative affect did not relate to any of the pain-related measures. In our setting, we focused only on the natural level of affect in a sample of healthy young adults, where negative affect was not particularly high and with a relatively low standard deviation as compared to positive affect.

Furthermore, it must be noted that the assessment of the natural level of affect with the PANAS might be considered as a measurement of personality traits; namely, that negative affect reflects the personality trait neuroticism and that positive affect reflects extraversion (Watson et al., 1992; for a review on state, personality traits, and personality change, see, e.g., Roberts, 2009). Thus, the relation of positive affect and pain sensitivity might reflect the well-documented extraversion pain sensitivity link (e.g., Lynn and Eysenck, 1961; Haslam, 1967).

The investigation of the relationship between pain, stress, and negative affect has a long tradition in pain research, with a strong focus on chronic pain conditions and negative affective states, such as depression and anxiety (Zautra et al., 2005). Studies applying various types of emotion evoking stimuli in healthy subjects (Meagher et al., 2001; Logan et al., 2003) were also not informative regarding the connection between pain perception and the natural changes of affect in the normal population. Our results show that, in a normal sample of young adults, the natural level of affect does not predict pain sensitivity.

Although the level of pain tolerance and threshold showed no association with cardiac IAc (hypotheses 1 and 2), we found interactions between sensitivity to pain and sex, i.e., males with higher level of interoceptive accuracy tend to have a higher pain threshold (hypothesis 5). These finding may be interpreted in the way that higher cardiac IAc (directly or indirectly) helps male participants to down-regulate their response to pain stimuli and thereby achieve higher pain threshold levels, whereas men with low cardiac IAc might rely more on response caution and report lower thresholds. The possible causes of this interaction will be discussed in more detail below.

Cardioceptive accuracy was not significantly associated with pain threshold. In fact, Bayesian correlation analysis indicated the superiority of the null hypothesis (BF < 0.1). Concerning cardiac IAc and tolerance (hypotheses 2), a weak association was revealed in the frequentist correlation analysis, which was not supported by the Bayesian analysis. The association also became non-significant after controlling for sex and affective state in the regression analysis. This lack of association between the two modalities (i.e., pain sensitivity and heartbeat perception scores) that are both considered interoceptive by some authors based on their common neurological pathways (Craig, 2015) is in accordance with the results of previous studies reporting the independence of pain perception and heartbeat perception (Werner et al., 2009; Ferentzi et al., 2017, 2018) and suggesting that interoceptive ability, as it is assessed by one indicator only, is not necessarily generalizable to other internal sensory channels (Ferentzi et al., 2017, 2018). There are some additional factors, however, to take into account. Recent findings confirmed that pain sensitivity might have a modality-specific characteristic, describing distinct pain pathways of visceral and somatic pain (Hockley et al., 2017). Additionally, experimentally induced pain might have different characteristics depending on the method applied to evoke it (e.g. Goodin et al., 2012). Accordingly, the possibility that some types of internal sensory channels are linked not only at the neurophysiological level (Craig, 2015) but also at the level of the individual performance on the sensory measures cannot be excluded. For future studies, more careful consideration is needed to explore which types of sensory tests might be more likely to be linked together.

Both pain threshold and tolerance were predicted by sex. Male participants showed considerably higher pain threshold and tolerance level than females (hypotheses 3 and 4). The theoretically expected interaction between sex and heartbeat perception was also supported by the data for pain threshold (hypothesis 5). In summary, the major determinants of pain sensitivity in healthy individuals appear to be sex and to a lesser extent the interaction between cardiac IAc and sex.

According to our findings, the associations between pain sensitivity, cardiac IAc, and sex might be more complicated than it was supposed in previous studies. Whereas women show no association between indicators of pain sensitivity and cardiac IAc, cardiac IAc and pain threshold are inversely related in men (i.e., those with a higher level of cardiac IAc show a higher pain threshold). These resultscould be explained by the idea of Pennebaker (Pennebaker and Roberts, 1992). He explained the better performance of males at interoceptive tasks in laboratory circumstances by their better abilities to detect internal signals. Women, on the other hand, take into consideration more external (environmental) cues when estimating their current inner state. As both ways are reliable under everyday conditions, the sex difference is not necessarily apparent outside the laboratory where external cues are not controlled for Pennebaker and Roberts (1992). In future studies, it would be worthwhile to investigate the mechanisms of interoceptive abilities in everyday situations.

Males usually show a higher pain threshold and tolerance level (Rhudy and Williams, 2005; Fillingim et al., 2009) as well as higher cardiac IAc (Katkin, 1985; Montoya et al., 1993; Grabauskaitė et al., 2017) (although, in the latter case, the findings are also controversial; Pennebaker, 1982; Mussgay et al., 1999; Pollatos and Schandry, 2004). Our results provide a more complete picture of the connection between the investigated variables. The association between higher pain threshold levels and higher cardiac IAc among men indicates that a sex-dependent extra mechanism might be involved. As pain can be characterized both by affective and by sensory components, it is possible that these can be regulated more effectively with higher inner focus, i.e., higher cardiac IAc among men. According to a brain imaging study, expectancies influence pain sensation (Atlas et al., 2010). A possible explanation of our result is that men with better cardiac IAc have higher expectancies concerning the pain stimulus, which makes them more prepared to get the pain stimulation that consequently becomes more manageable. A similar suggestion has been made by previous studies investigating interoception and emotion regulation (Füstös et al., 2013).

The current study has several limitations. First of all, the validity of the mental heartbeat tracking task by Schandry (1981) has beenquestioned by numerous authors (Ring et al., 2015; Desmedt et al., 2018; Murphy et al., 2018; Ring and Brener, 2018), and there is an ongoing debate about its validity (Zamariola et al., 2018; Ainley et al., 2020; Corneille et al., 2020; Zimprich et al., 2020). According to a recent study, the instructions allowing estimation could lead to different results than a stricter version of the instructions (Desmedt et al., 2020). However, in the original protocol of Schandry (1981), estimation was also allowed. The rationale of this is that heartbeats are weak sensations, and as such (similarly to ambiguous external sensations), estimation can reflect indeed a true but ambiguous sensation. Nevertheless, in the light of the recent findings and the debate mentioned above, more investigation is needed on the exact influence of the type instruction given.

Additionally, as pain has been stimulated electrically, it might not be generalizable to pain sensitivity induced differently, as it has been mentioned above. For example, a comparison of the experimental pain stimulation induced by four types of laser showed differences between the stimulation types (Svensson et al., 1991). The characteristics of the investigated sample (e.g., healthy young individuals) also limit the generalizability of the findings. Finally, the conclusion introduced above is valid only if the assumed medium effect size holds.

## Data Availability Statement

The raw data supporting the conclusions of this article will be made available by the authors, without undue reservation.

## Ethics Statement

The studies involving human participants were reviewed and approved by Ethics Committee of Ulm University. The patients/participants provided their written informed consent to participate in this study.

## Author Contributions

MG, SAM-L, CM, and OP contributed to the conception and design of the study. MG contributed to the assessment of data, while EF and FK processed the data and performed the statistical analyses. EF wrote the first draft of the manuscript. MG, FK, and OP wrote sections of the manuscript. All authors read and commented on the final version of the manuscript.

## Conflict of Interest

The authors declare that the research was conducted in the absence of any commercial or financial relationships that could be construed as a potential conflict of interest.
